# Cdc42 Effector Protein 3 Interacts With Cdc42 in Regulating Xenopus Somite Segmentation

**DOI:** 10.3389/fphys.2019.00542

**Published:** 2019-05-07

**Authors:** Mary Kho, Hongyu Shi, Shuyi Nie

**Affiliations:** ^1^School of Biological Sciences, Georgia Institute of Technology, Atlanta, GA, United States; ^2^Petit Institute for Bioengineering and Bioscience, Georgia Institute of Technology, Atlanta, GA, United States; ^3^Integrated Cancer Research Center, Georgia Institute of Technology, Atlanta, GA, United States

**Keywords:** Cdc42, Cdc42ep3, somitogenesis, somite segmentation, epithelialization, *Xenopus*

## Abstract

Somitogenesis is a critical process during vertebrate development that establishes the segmented body plan and gives rise to the vertebra, skeletal muscles, and dermis. While segmentation clock and wave front mechanisms have been elucidated to control the size and time of somite formation, regulation of the segmentation process that physically separates somites is not understood in detail. Here, we identified a cytoskeletal player, Cdc42 effector protein 3 (Cdc42ep3, CEP3) that is required for somite segmentation in *Xenopus* embryos. *CEP3* is specifically expressed in somite tissue during somite segmentation. Loss-of-function experiments showed that CEP3 is not required for the specification of paraxial mesoderm, nor the differentiation of muscle cells, but is required for the segmentation process. Live imaging analysis further revealed that CEP3 is required for cell shape changes and alignment during somitogenesis. When CEP3 was knocked down, somitic cells did not elongate efficiently along the mediolateral axis and failed to undertake the 90° rotation. As a result, cells remained in a continuous sheet without an apparent segmentation cleft. CEP3 likely interacts with Cdc42 during this process, and both increased and decreased Cdc42 activity led to defective somite segmentation. Segmentation defects caused by Cdc42 knockdown can be partially rescued by the overexpression of CEP3. Conversely, loss of CEP3 resulted in the maintenance of high levels of Cdc42 activity at the cell membrane, which is normally reduced during and after somite segmentation. These results suggest that there is a feedback regulation between Cdc42 and CEP3 during somite segmentation and the activity of Cdc42 needs to be fine-tuned to control the coordinated cell shape changes and movement required for somite segmentation.

## Introduction

Somitogenesis is an essential step during vertebrate development. During somitogenesis, the paraxial mesoderm is partitioned into bilaterally symmetric blocks called somites, which later give rise to the dermis, skeletal muscles, and axial bones including vertebrae and ribs (Christ and Ordahl, 1995; Pourquie, 2001; Saga and Takeda, 2001). Somitogenesis is critical for the establishment of the segmented body plan of all vertebrates. This step is important not only for the segmented structure of axial bones and muscle, but also for correct patterning of the peripheral nervous system and the blood vessels. As a result, defective somite segmentation leads to severe defects in the formation and alignment of vertebrae and ribs, as well as defective vasculature, such as occurs in spondylocostal dysostosis and Alagille syndrome (Shifley and Cole, 2007; Turnpenny, 2008).

Somite segmentation takes place periodically and regularly in an anterior to posterior sequence, which is orchestrated by several coordinated signaling events. Notch pathway genes are expressed in pre-somitic mesoderm (PSM) in a cyclic manner, which determines the speed of segmentation (Sato et al., 2002). At the same time, the intersection (or wave front) of the posterior-anterior gradient of fibroblast growth factor (FGF) and Wnt and the anterior-posterior gradient of retinoic acid (RA) capacitates cells to respond to the Notch-mediated segmentation clock, thus determining where segmentation takes place (Pourquie, 2003; Dubrulle and Pourquie, 2004; Moreno and Kintner, 2004). While the segmentation clock and wave front mechanism has been established to control the timing and space of somite segmentation, the cellular events that mediate the separation of somites are less understood. A study in chick embryos demonstrated that Rho GTPases Cdc42 and Rac1 are involved in the mesenchymal-to-epithelial transition (MET) during somitogenesis (Nakaya et al., 2004). Somitomeres in birds and mammals have an outer epithelial layer and a mesenchymal interior. Nakaya et al. showed that Cdc42 activity needs to be suppressed in boundary cells for them to become epithelial, which is critical for the formation of distinct morphological boundaries between somitomeres. When cells were forced to express high levels of Cdc42, they moved to the center of the somite, where mesenchymal cells reside (Nakaya et al., 2004). In contrast, a moderate level of Rac1 activity needs to be maintained for MET to take place, and it interacts with the transcription factor Paraxis during this process (Nakaya et al., 2004). In the frog *Xenopus laevis*, somite formation is slightly different in that the somites do not become epithelialized (Youn and Malacinski, 1981a,b; Keller, 2000; Afonin et al., 2006). Instead, cells elongate mediolaterally and align with the same orientation. Then, a thin but discrete fissure appears between the developing somitomeres. This intersomitic boundary becomes more evident as cells within each somite block undergo a rearrangement and rotation event to adopt an anterior-posterior orientation. At the same time, matrix deposition and assembly occurs around each somite block, physically separating the somites and supporting the alignment of the myotome. A recent report showed that although there is no MET process during *Xenopus* somitogenesis, Paraxis is still required for regulating cell-cell adhesion during this process (Sanchez and Sanchez, 2015). Whether Cdc42 also plays a conserved role in *Xenopus* somitogenesis and which signal mediates its activity during somitogenesis is still unclear.

In this study, we identified an effector protein for Cdc42, Cdc42 effector protein 3 (Cdc42ep3 or CEP3), which is specifically expressed in the developing somite. CEP3 belongs to a small family of Cdc42 effector proteins, also named binders of Rho GTPases (Borgs) (Joberty et al., 1999; Farrugia and Calvo, 2016). There are 5 CEPs in vertebrates, and their function in development is not well-understood. In mice, CEP1 (Borg5) enhances trophectoderm differentiation by promoting the sorting of trophectodermal cells to the outer layer of the blastocyst (Vong et al., 2010). Later in microvascular angiogenesis, CEP1 regulates directional migration of endothelial cells (Liu et al., 2014). We have recently reported that CEP1 interacts with Cdc42 to regulate actin organization during neural crest cell migration in frog embryos (Cohen et al., 2018). CEP2 (Borg1) has also been studied in *Xenopus* embryos and it promotes the cell-cell adhesion of non-neural ectoderm and the involution of mesoderm during gastrulation (Nelson and Nelson, 2004). CEP3 (Borg2) has not been studied in animal development and is best studied in cancer-associated fibroblasts (CAFs). CEP3 stabilizes actin stress fibers and septin networks in CAFs and is critical for CAFs to generate and sense forces. In addition, CEP3 is required for CAFs to remodel extracellular matrix, promote angiogenesis, and to promote cancer cell growth and invasion (Calvo et al., 2015; Farrugia and Calvo, 2017). While there is little knowledge of CEP3 in development or disease, human genome-wide association studies suggest that CEP3 is associated with adult height, muscular hypotonia, and joint laxity (Lango Allen et al., 2010).

Here, we investigated the role of CEP3 during *Xenopus* somitogenesis. Our results show that CEP3 is required for somitogenesis in *Xenopus* embryos. Loss of CEP3 in paraxial mesoderm led to defective somite segmentation, without affecting paraxial mesoderm specification or myogenesis. Using Wilson explants to visualize somite segmentation in live tissue, we further showed that CEP3 is not only required for somite segmentation, but also for somitic cells to elongate mediolaterally. Cdc42 activity needs to be tightly controlled during somitogenesis, and CEP3 not only acts downstream of Cdc42 to regulate this process, but also provides feedback regulation to Cdc42 activity. During somite segmentation, active Cdc42 is normally reduced at the cell membrane. However, when CEP3 was knocked down, active Cdc42 was maintained at the cell membrane. These results demonstrate that in *Xenopus* embryos where somitic cells do not become epithelialized, Cdc42 and its effector protein CEP3 still play an essential role in the segmentation process.

## Materials and Methods

### Embryo Manipulations, Morpholino Oligomers, and RNA Preparation

*Xenopus laevis* embryos, both pigmented and albino, were obtained and staged as described by Nieuwkoop and Faber's Normal Table of *X. laevis*. The embryos were microinjected with capped RNAs or morpholino oligomers (MO) during early cleavage stages. standard control MO (Gene Tools, Philomath, OR) and Cdc42 effector protein 3(CEP3)-MO (5′- GGAATGAAATACGCAGATGTCAGAT−3′) that hybridizes to −32 to −8 position relative to the translational start site of *Xenopus CEP3* (GenBank Accession No. NM_1095138) were used in the study. GFP-wGBD was a gift from William Bement (Addgene plasmid # 26734). pcDNA3-EGFP-Cdc42-T17N and pcDNA3-EGFP-Cdc42-Q61L were gifts from Gary Bokoch (Addgene plasmid #12976/ #12986). RNAs of nuclear beta-galactosidase (nβGal), membrane-tethered EGFP, GFP-wGBD, Cdc42(T1N), Cdc42(Q61L), and CEP3 were synthesized with linearized templates using SP6 polymerase (Ambion mMessage mMachine Kit). To target the paraxial mesoderm, mRNA or MO was injected into the lateral marginal zone at the 2-cell stage. Five to ten nanograms of MO or 0.05–0.3 ng of RNA was injected into one side of the embryo as indicated in each experiment. All experimental procedures were performed according to the USDA Animal Welfare Act Regulations and had been approved by the Institutional Animal Care and Use Committee, in compliance of the Public Health Service Policy.

### Red-Gal Staining, *in situ* Hybridization, and Immunohistochemistry

For lineage tracing, embryos co-injected with nβGal were fixed at the desired stage for half an hour in the fixative MEMFA and stained with the Red-Gal substrate (Research Organics) until they turned red. The embryos were refixed for 2 h in MEMFA, and stored in methanol before *in situ* hybridization was performed. Whole-mount *in situ* hybridization was performed as previously described (Cohen et al., 2018). Antisense probes for *cdc42ep3, tbxt* (*Xbra*, NM_001091696)*, mef2d* (NM_001096493), *myf5* (NM_001101779), *myf6* (NM_001088008), *myod1* (NM_001087823), and *myog* (NM_001085857) were synthesized with T7 RNA polymerase with linearized plasmids. Immunohistochemistry was performed as previously described (Cohen et al., 2018) against muscle antibody 12/101 (DSHB).

### Somite (Wilson) Explant Culture and Microscopy

Wilson explants were isolated from embryos at stage 13–14 as previously described (Wilson et al., 1989). Briefly, embryos were cut open along the lateral walls of the archenteron, and the endoderm of the archenteron roof was carefully peeled away, exposing the notochord and paraxial mesoderm. The explants were then gently pressed down onto fibronectin (FN, 20 μg/ml in PBS)-coated dishes by coverslips supported by silicon grease in Danilchik media (Wilson et al., 1989). Static images or time-lapse movies were acquired using the PerkinElmer Spinning Disc confocal microscope with 10x and 20x objectives. To analyze cell shape changes, the ImageJ circularity Plugin was used. To analyze the distribution of active Cdc42, intensity plots of wGBD-GFP across the medial-lateral axes of cells were analyzed in ImageJ. Both the length of the cell and the signal intensity (relative to average intensity across the same cell) was normalized for plotting.

## Results

### CEP3 Is Specifically Expressed in the Developing Somite

**Figure 1:**
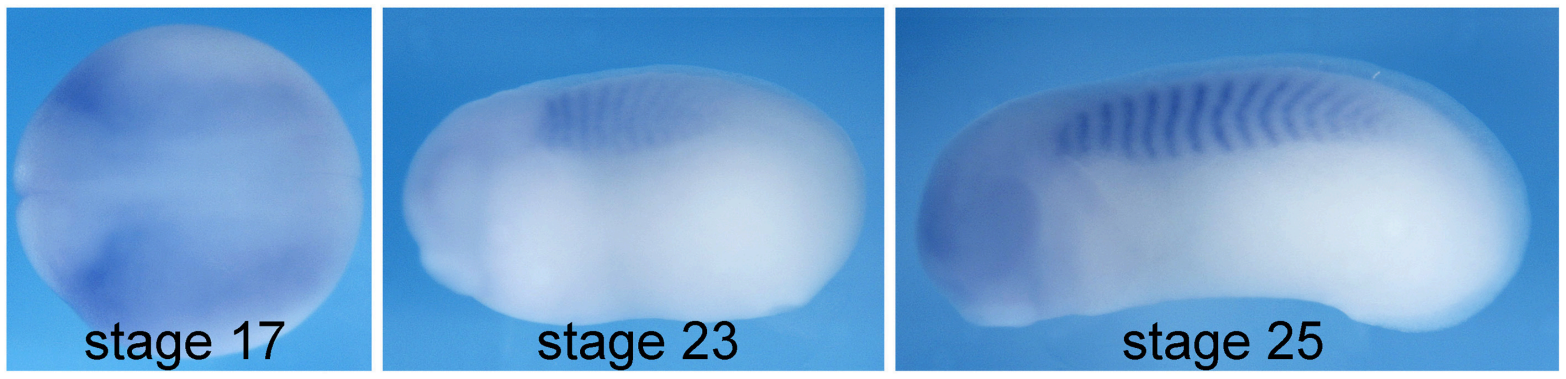
Expression of *cdc42ep3* during *Xenopus* embryogenesis. *In situ* hybridization analysis shows that *cdc42ep3 (CEP3)* is specifically expressed in paraxial mesoderm and the developing somite. At stage 17, *CEP3* is broadly expressed in the paraxial mesoderm before somite segmentation takes place. During somite segmentation, *CEP3* is specifically expressed in segmented somites.

Cdc42 is involved in various cell and tissue morphogenesis events, and consistently, it is broadly expressed during embryonic development (Choi and Han, 2002). CEPs, unlike many other effector proteins for Cdc42 (e.g., N-WASP and PAK1), are only evolved in vertebrates. Therefore, it is likely that they play a role in the development of vertebrate-specific structures, such as somites. To determine whether CEP3 mediates Cdc42 activity in somitogenesis, we first examined the expression of *CEP3* by *in situ* hybridization analysis. As shown in Figure 1, at neurula stages around the onset of somitogenesis, *CEP3* is weakly expressed throughout the paraxial mesoderm. Later at tailbud stages, *CEP3* is specifically expressed in the developing somites, but not in the pre-somitic mesoderm (PSM) posterior to the forming somite. The expression pattern of *CEP3* suggests that it may play a role in somitogenesis.

### CEP3 Is Required for Somite Segmentation

**Figure 2:**
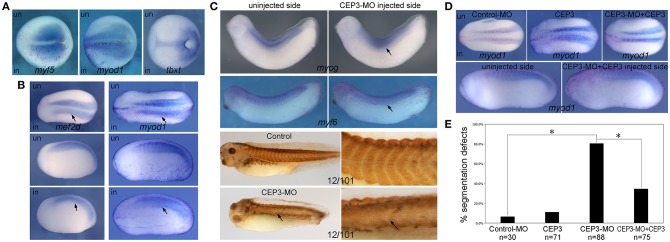
Cdc42ep3-MO affected the expression of somitic genes. **(A–C)** Embyros were injected with 10 ng of CEP3-MO on one side, and the expression of somitic genes were compared between injected and uninjected sides. At the late gastrula stage (stage 15), CEP3-MO did not affect the expression of mesodermal genes m*yf5* or *myod1* or pan-mesodermal gene *tbxt (Xbra)*. At the early neurula stage (stage 22), the expression of *mef2d* and *myod1* shows a segmented pattern in the uninjected side, but not in the CEP3-MO-injected side (arrows). At late tailbud stages, the segmented expression pattern, but not the overall expression level of myogenic genes *myog* and *myf6* was affected by CEP3-MO. Similar segmentation defect was observed in embryos stained with 12/101 antibody. **(D)** While 0.3ng CEP3 alone did not induce obvious segmentation defect, coinjection of CEP3 with CEP3-MO partially rescued the segmentation defects. Dorsal view (**A**, top panels in **B**, and top panels in **D**) or lateral view (bottom panels in **B,C**, and bottom panels in **D**, showing both sides of the same embryo) of the embryos was shown, with anterior to the left. **(E)** The percentage of defective embryos were summarized in the bar graph. χ^2^ test was performed and both CEP3-MO mediated knockdown and CEP3 rescue led to significant difference in the phenotype. ^*^P < 0.01.

Since *CEP3* is specifically expressed in developing somites, we next determined whether CEP3 is required for somite development and in which step CEP3 is required. Loss-of-function experiments were performed with a translation-blocking morpholino oligomer (MO) against *CEP3*. Ten nanograms of CEP3-MO together with a lineage tracer nuclear beta-galactosidase (nβGal) was injected into one side of 2-cell stage embryos, leaving the contralateral side as an internal control. Embryos were fixed at gastrula stage (stage 13), early tailbud stage (stage 22–23), and late tailbud stage (stage 28–30), and subject to *in situ* hybridization against different mesodermal and myogenic genes. During gastrulation, the paraxial mesoderm is specified by gradients of morphogens such as bone morphogenetic proteins (BMPs), FGFs, Wnt, and Noggin. At this stage, CEP3-MO did not affect the expression of a pan-mesodermal gene *tbxt* (*Xbra*) (Figure 2A; *n* = 9/9). The somite in *Xenopus* embryos primarily gives rise to myotome tissue, and some myogenic genes are already expressed in paraxial mesoderm at gastrula stages. When CEP3 was knocked down by CEP3-MO, the expression of myogenic genes *myf5* and *myod1* was not affected (Figure 2A; *n* = 16/16 total). These results indicate that CEP3 is not required for the specification of paraxial mesoderm.

At early tailbud stages when somite segmentation is underway, the first wave of myogenesis has occurred to generate differentiated myotome (Della Gaspera et al., 2012). *Mef2d* and *myod1* are expressed in a segmented pattern in the developing somite and uniformly in the PSM. When CEP3 was knocked down by CEP3-MO, the segmented expression pattern of *mef2d* and *myod1* was lost (arrows in Figure 2B; *n* = 17/22, 28/31, respectively). The expression levels of both genes were relatively unchanged, but they were expressed in a diffused and continuous manner. This result indicates that CEP3 is required for somite segmentation. Later in development, the somite is further differentiated, and more muscle-specific transcription factors are expressed. *Myog* is expressed continuously in the central domain of each somite, and in a segmented manner in the dorsal and ventral aspect of the somite. When CEP3 was knocked down, the expression level of *myog* was unaffected, but the segmented pattern was lost (Figure 2C; *n* = 12/17). Similarly, the segmented expression of *myf6* and muscle marker 12/101 were disrupted by CEP3-MO (*n* = 9/12, 5/6, respectively). Unlike the chevron shaped somites seen in control embryos, somitic tissue appeared in a disorganized and continuous form. These results confirmed that somite segmentation, but not myogenesis was inhibited by the loss of CEP3. The expression pattern of myogenic genes at late developmental stages also indicated that CEP3-MO did not cause a delay in somite segmentation, but rather inhibited segmentation. When CEP3 RNA without 5′UTR (therefore cannot be blocked by CEP3-MO) was coinjected with CEP3-MO, the segmentation defect was partially rescued (Figure 2D; *n* = 8/27, 18/48 with segmentation defect for *mef2d* and *myod1*, respectively), suggesting that the segmentation defect is specific to the loss of CEP3. Results obtained from the above *in situ* hybridization experiments were pooled together and summarized in Figure 2E. CEP3 knockdown increased the percentage of defective embryos significantly, which in turn was rescued by the addition of CEP3 significantly.

### CEP3 Regulates Cell Morphology and Rotation During Somite Segmentation

**Figure 3:**
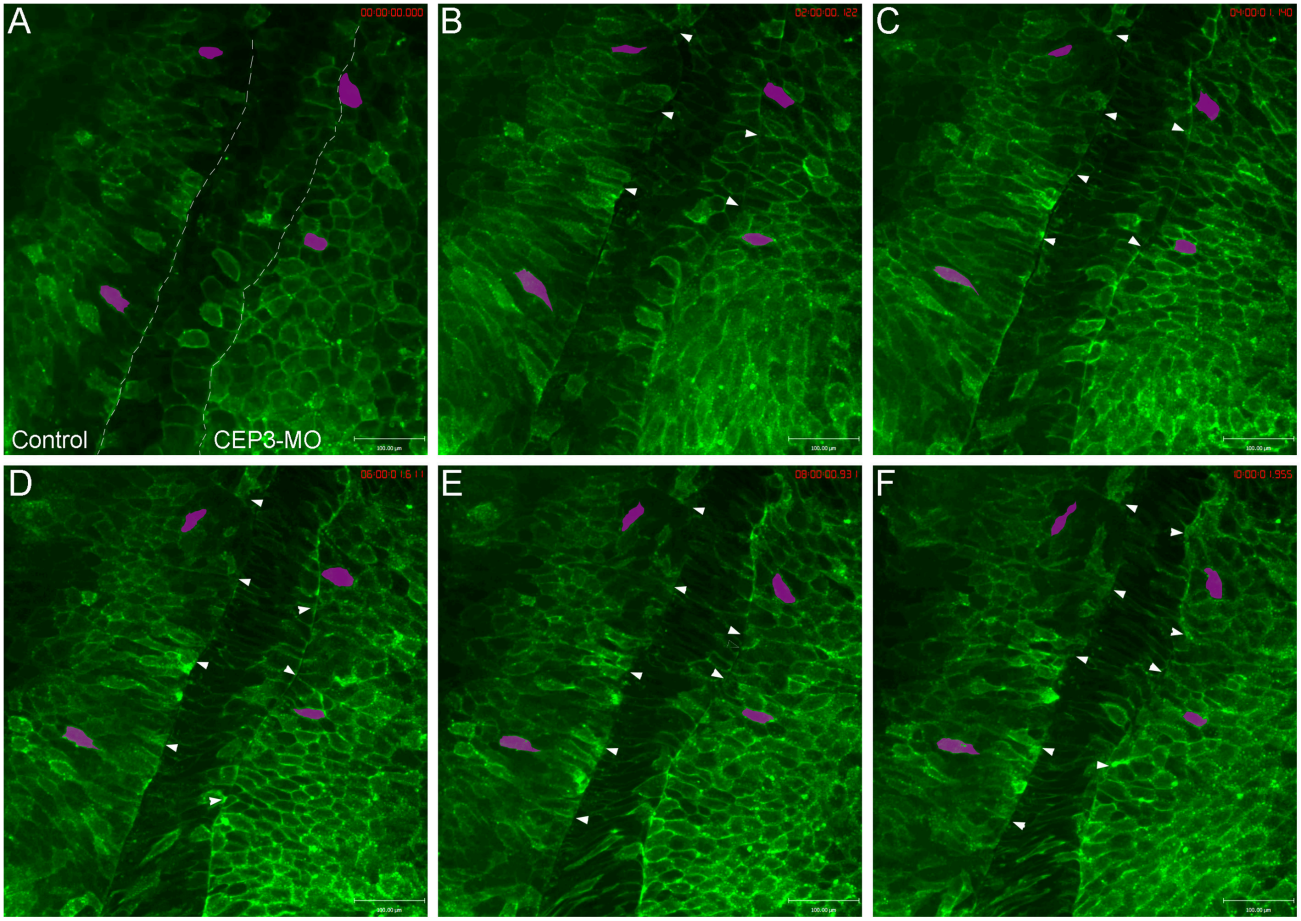
Loss of Cdc42ep3 disrupted somite segmentation and cell shape changes. Wilson explants were dissected from embryos receiving membrane-tethered EGFP (EGFP-CAAX) on one side, and CEP3-MO plus EGFP-CAAX on the other side, and the segmentation process was followed by time-lapse microscopy. **(A–F)** Time frames at 2-h intervals. The dashed lines in **(A)** marks the boundaries between pre-somitic mesoderm and the notochord. Arrowheads marked segmentation furrows. Two cells on each side of the explant were followed by magenta shade. CEP3-MO not only impaired somite segmentation, but also affected the mediolateral elongation of cells. Scale bar = 100 μm.

Since somite segmentation is a dynamic process, we next used live imaging to document the segmentation process and to determine how CEP3-MO affected somite segmentation. Wilson explants were prepared to expose the ventral surface of the notochord and paraxial mesoderm, yet preserve tissue morphogenesis for many hours (Wilson et al., 1989). One side of the embryo was injected with membrane-tethered EGFP to reveal the shape of cells, and the other side of the embryo was injected with membrane-tethered EGFP together with 5 ng of CEP3-MO. This reduced dose of MO is used to generate a milder segmentation defect to allow for the analysis of various aspects of cell morphology and movements. The Wilson explant was imaged at 10-min intervals for around 10 h, from roughly embryonic stage 17 to stage 22 (Supplementary Movie 1). Time frames from one representative movie are shown in Figure 3. In contrast to the distinct segmentation fissures on the control side (marked by arrowheads), there were fewer segmentation fissures that were obvious on CEP3-MO-injected side. A segmentation fissure was sometimes observed earlier, but disappeared at a later time points (arrowheads). We did not observe the regular 50-min pace of somite formation in our movies, which was likely caused by two reasons. First, the dissected explant cannot completely resemble the intact embryo, especially as somitogenesis involves large-scale tissue rearrangements. Second, at the same axial level, somitogenesis progresses in a dorsal to ventral sequence (Afonin et al., 2006). Therefore, somitogenesis at the ventral surface (observed in Wilson explants) may display some delay comparing to that at the dorsal surface (revealed by whole-mount immunohistochemistry). Nevertheless, our observations confirmed that CEP3 is required for somite segmentation.

Besides a failure to organize into distinct somitomeres, cells deprived of CEP3 also appeared different in shape compared to control cells. On the control side, cells elongated mediolaterally before segmentation took place. After segmentation, cells maintained their elongated shape and gradually rotated 90° to adopt an anterior-posterior orientation (see magenta-shaded cells in Figure 3). In contrast, cells on CEP3-MO-injected side were much rounder in shape and occasionally even elongated along the anterior-posterior axis. While they do elongate mediolaterally over time, the cells never adopt the same elongated shape as control cells. The shapes of cells were quantified by the ImageJ circularity plugin (circularity = 4pi (area/perimeter^2^), with circularity = 1 for a perfect circle and circularity = 0 for a straight line). Over 100 cells were analyzed from 3 explants, with a similar number of cells picked on control and experimental sides at each axial level (before and after segementation, but not undergo rotation). While control cells have an average circularity of 0.454 ± 0.10, CEP3-MO cells have an average circularity of 0.698 ± 0.09. This difference was statistically significant (*p* = 2.5E-15; student *t*-test).

After the initial segmentation, cells start to rotate from a mediolateral orientation to an anteroposterior orientation. Possibly due to the isolation of the Wilson explant from the intact embryo, we only observed the rotation events in the first 2–3 somites on the control side. When CEP3 was knocked down, this rotation process was also hindered. While this could result from impaired segmentation in the first place, inadequate somite rotation may also exacerbate the segmentation defect. Taken together, CEP3 may regulate cell shape changes and rotation to control somite segmentation.

### CEP3 Interacts With Cdc42 in Somite Segmentation

**Figure 4:**
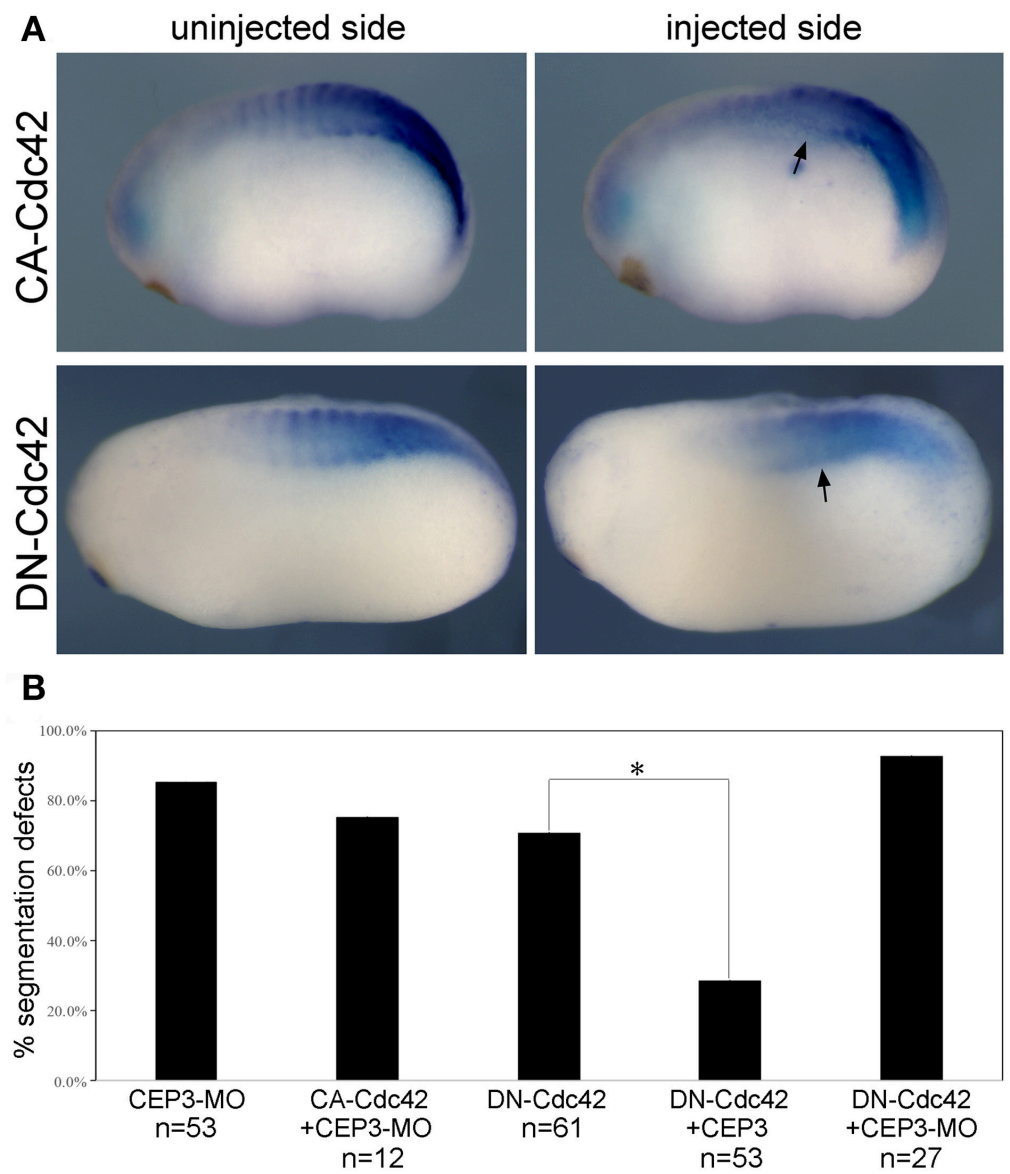
Cdc42 needs to be tightly controlled during somite segmentation. **(A)** 0.05 ng of constitutively active (CA) or dominant negative (DN) Cdc42 was injected into one side of the embryo, and their effect on somite segmentation was examined by the expression of m*yod1*. Comparing to the uninjected control side, the segmented expression pattern of *myod1* was lost on the injected side (arrows), suggesting that both increased and decreased activity of Cdc42 will affect somite segmentation. **(B)** CA-Cdc42 was co-injected with CEP3-MO, and CEP3 or CEP3-MO was co-injected with DN-Cdc42 to determine whether they can rescue segmentation defects caused by CEP3 or Cdc42 knockdown. The percentage of embryos with segmentation defect was summarized in the bar graph. χ^2^ test indicates that CA-Cdc42 failed to rescue segmentation defects caused by CEP3-MO (*p* = 0.52), but CEP3 significantly rescued segmentation defects caused by DN-Cdc42 (^*^p < 0.01). Coinjection of CEP3-MO with DN-Cdc42, on the other hand, further inhibited somite segmentation (*p* = 0.02 when comparing with DN-Cdc42 alone).

The activities of CEP3 in regulating cell shape changes and tissue arrangements likely result from its interaction with Cdc42. Cell culture studies have demonstrated that CEPs can mediate the activity of Cdc42 in the formation of membrane protrusions (Joberty et al., 1999, 2001; Hirsch et al., 2001; Calvo et al., 2015). In chick embryos, Cdc42 has been reported to control somite segmentation through regulating the MET process (Nakaya et al., 2004). In that case, boundary cells need to turn down Cdc42 to become epithelial so that individual somitomeres can form. To determine if CEP3 interacts with Cdc42 during *Xenopus* somite segmentation, we first examined the function of Cdc42 in this process. To avoid interrupting GTPase-independent activity of Cdc42, we expressed dominant negative (DN-) and constitutively active (CA-) Cdc42 to alter the activation level of Cdc42 (Nalbant et al., 2004). DN-Cdc42 carries a point mutation (T17N) that prevents it from binding to GTP, so that it is always in the inactive, GDP-bound, state. Moreover, it sequesters guanine nucleotide exchange factors, thus also preventing the activation of endogenous Cdc42. Conversely, CA-Cdc42 [Cdc42(Q61L)] prevents GTPase activating protein-mediated GTP hydrolysis, therefore remaining active. Fifty picograms of DN-Cdc42 or CA-Cdc42 was expressed in paraxial mesodermal cells, and their effect on somite segmentation was examined by *in situ* hybridization against myogenic genes *mef2d* and *myod1* (numbers were summarized together). Both DN-Cdc42 and CA-Cdc42 disrupted the segmentation expression pattern of myogenic genes (Figure 4A; *n* = 43/61, 7/23, respectively), suggesting that tightly controlled Cdc42 activity is required for proper segmentation process. A rescue experiment was performed next to determine whether CEP3 mediates Cdc42 during somite segmentation. While co-expression of CA-Cdc42 with CEP3-MO could not rescue segmentation defects caused by CEP3-MO, co-expression of CEP3 RNA with DN-Cdc42 partially rescued segmentation defects caused by DN-Cdc42 (Figure 4B). These results suggest that CEP3 acts downstream of Cdc42 in somitogenesis.

**Figure 5:**
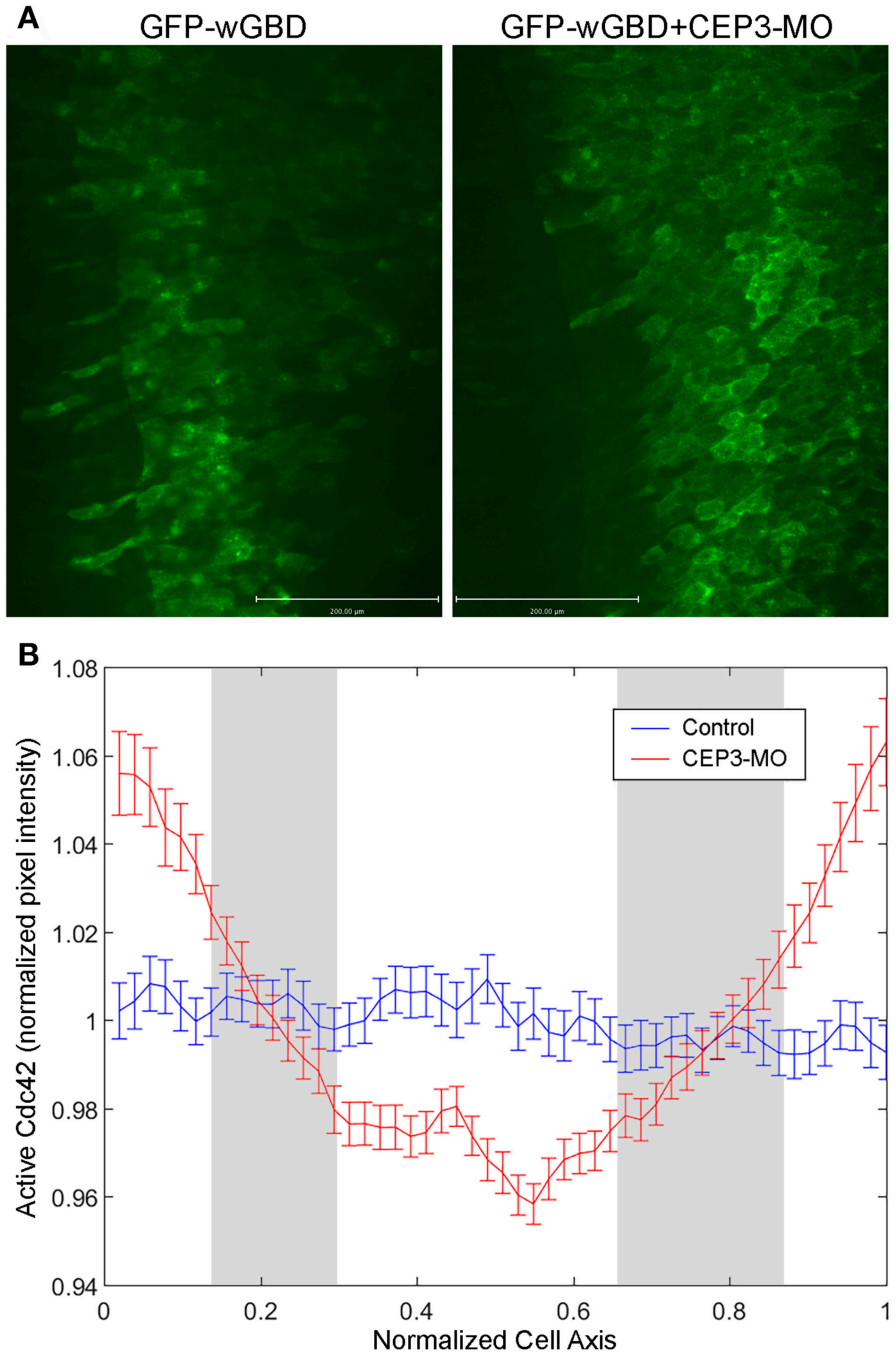
CEP3-MO regulates Cdc42 activity in the developing somite. GFP-wGBD that binds to active Cdc42 was expressed in the paraxial mesoderm alone or together with CEP3-MO. **(A)** While GFP-wGBD was largely internalized and distributed in a diffused manner in control somitic cells, it was localized to the cell membrane in CEP3-MO-injected cells, reflecting a higher level of Cdc42 activity. **(B)** An intensity plot of GFP-wGBD across the mediolateral axis of cells was generated. There was a significantly higher level of GFP-wGBD at the cell periphery in CEP3-MO-injected cells and a significantly lower level of GFP-wGBD in the center of CEP3-MO-injected cells. Error bars: standard error of the mean. Student *t-*test was performed, and the gray area marks places where *p* > 0.01.

In neural crest cells, we previously showed that CEP3 paralog CEP1 provides feedback regulation of Cdc42 by regulating the subcellular localization of active Cdc42 (Cohen et al., 2018). Here, we examined whether CEP3 also controls the localization of active Cdc42 during somitogenesis. We used GFP-wGBD [GFP fusion with the Cdc42-binding domain of N-WASP; (Benink and Bement, 2005)], which specifically binds to active Cdc42, to examine the localization of active Cdc42 in somitic cells. One side of the embryo was injected with 0.1 ng of GFP-wGBD alone, and the other side was injected with 0.1 ng of GFP-wGBD together with 5 ng of CEP3-MO. Wilson explants were prepared, and the localization of active Cdc42 was analyzed at early tailbud stage (stage 20) and compared between cells on both sides of the explant. On the control side, there were low levels of active Cdc42 at the cell membrane. Instead, the active-Cdc42 reporter was often observed inside the cell in a punctate manner, possibly reflecting reporters that failed to bind to Cdc42. In contrast, CEP3-MO-injected cells maintained a higher level of active Cdc42 on their surface (Figure 5A, explants from two embryos that received similar level of GFP-wGBD were shown). Although each cell may receive a different amount of the reporter, the ratio of reporter proteins on cell membrane should correlate with the level of active Cdc42. Therefore, to quantitate the levels of active Cdc42, intensity plots across the long axis of cells were generated and analyzed (Figure 5B). Over 170 cells from 20 Wilson explants were analyzed. Since the cells were of different sizes and have received a slightly different amount of GFP-wGBD due to injection, both the length of the cells and the pixel intensity of GFP-wGBD was normalized. To assess the ratio of wGBD reporter on the cell membrane, we normalized the signal intensity by comparing the actual pixel intensity at each location in a cell to the average pixel intensity across the same cell. Our result showed that GFP-wGBD distributed evenly across the mediolateral axis of control cells, possibly reflecting a low level of Cdc42 activity. The loss of CEP3, however, lead to high levels of GFP-wGBD at the cell periphery, suggesting a higher level of Cdc42 activity. This change in GFP-wGBD distribution was significant (white areas in Figure 5B are places where *p* < 0.01). This result suggests that Cdc42 activity needs to be decreased during somite segmentation and this partly explains why CA-Cdc42 also affected somite segmentation. Moreover, CEP3 may provide a negative feedback to Cdc42 to fine-tune Cdc42 activity in regulating somite segmentation.

## Discussion

### CEP3 in Somite Segmentation

In this study, we showed that CEP3 is required for somite segmentation in *Xenopus* embryogenesis. We think that CEP3 is involved in processes after the establishment of the segmentation clock since the expression pattern of *EphA4*/*EphrinB2* downstream of the segmentation clock was not affected (data not shown) (Watanabe et al., 2009). Our live imaging analysis of Wilson explants suggests three possible mechanisms of how CEP3 regulates somite segmentation. First, CEP3 may control segmentation by regulating cell shape changes. PSM cells usually elongate mediolaterally before segmentation takes place, and this was inhibited by CEP3-MO (Figure 3). Whether such elongation is required for somite segmentation is unclear, but it may enable cell-matrix interactions that promote cell alignment. Around the time of somite segmentation, PSM cells reach a long columnar shape across the entire paraxial mesoderm, contacting the extracellular matrix between the paraxial mesoderm and the notochord. This matrix interaction may also help maintain the elongated cell shape. The mediolateral elongation is not restricted to the somitic cells. It mediates a general convergent extension movement undertaken by the entire axial and paraxial mesoderm. Whether CEP3 plays a role in convergent extension, or whether it interacts with signals such as the planar cell polarity pathway that controls convergent extension remains to be tested.

The second possible way through which CEP3 regulates somite segmentation is the regulation of matrix deposition. Both fibronectin and laminin matrix are deposited and assembled at the intersomitic boundaries, as well as boundaries between notochord and the PSM. This extracellular matrix not only protects the integrity of each somitomere but also plays essential roles in somitic cell elongation, alignment, and rotation (Kragtorp and Miller, 2007; Hidalgo et al., 2009). Given that the expression of *CEP3* in somitic cells increases after somite segmentation and that intersomitic fissures can form but are not maintained at the loss of CEP3, it is possible that CEP3 also influences matrix deposition and assembly around the somitomeres. In endothelial cells, Rac1 and Cdc42 levels influence fibronectin matrix assembly during vascular morphogenesis (Fernandez-Sauze et al., 2009). Whether CEP3 regulates matrix assembly during somite segmentation through its interaction with Cdc42 remains to be elucidated.

Lastly, to maintain somite segmentation, CEP3 could also regulate cell rotation and alignment. The rotation of somitomeres has also been observed in zebrafish (Hollway et al., 2007), possibly correlating to an adapted program in swimming embryos with somites mostly comprised of myotome fibers. After rotation, cells align anteroposteriorly, contacting both intersomitic boundaries, and therefore also contribute to the stability of the somite. Afonin et al. showed that the rotation event associates with increased filopodia protrusions (Afonin et al., 2006). Since Cdc42 is critical in filopodia formation, it is possible that CEP3 interacts with Cdc42 to promote the protrusive activity of rotating cells, thus regulating the rotation event. In cell cultures, CEPs have been reported to be involved in cell protrusions. CEP1 induces membrane ruffling in Cos-7 cells and induces long actin-based protrusions in NIH 3T3 fibroblasts (Burbelo et al., 1999). In our study, CEP3 altered the activity of Cdc42, which is important for actin polymerization at the cell periphery for membrane protrusions. Whether CEP3 mediates Cdc42 in filopodia formation in somite cells remains to be examined.

### Interactions Between CEP3 and Cdc42

CEP3 is an effector protein for Cdc42; therefore, its role in somite segmentation likely involves its interaction with Cdc42. Our results suggest that there is cross-regulation between CEP3 and Cdc42. CEP3 acts downstream of Cdc42 such that segmentation defects caused by DN-Cdc42 can be partially rescued by CEP3 RNA, but segmentation defects caused by CEP3-MO cannot be rescued by CA-Cdc42 or DN-Cdc42. Conversely, CEP3 also modulates the activity of Cdc42. When CEP3 is knocked down by CEP3-MO, active Cdc42 on the plasma membrane is maintained, suggesting a feedback regulation between CEP3 and Cdc42. This is similar to our recent report that in neural crest cells, CEP3 paralog CEP1 provides feedback regulation of Cdc42 by regulating the subcellular localization of active Cdc42 (Cohen et al., 2018). We currently do not know through which mechanism CEP3 regulates Cdc42 activity. Whether CEP3 regulates the activation of Cdc42 through interacting with a GEF or GAP, or regulates the subcellular localization of Cdc42 through their physical interaction remains to be investigated.

## Ethics Statement

This study was carried out in accordance with the recommendations of USDA Animal Welfare Act Regulations and Public Health Service Policy. The protocol was approved by the Institutional Animal Care and Use Committee.

## Author Contributions

HS performed the initial characterization of loss of CEP3 phenotype, which is confirmed by MK. MK further examined the activity of CEP3 in somite segmentation with live imaging and examined the interaction between CEP3 and Cdc42. SN designed and guided the experiments, and prepared the manuscript.

### Conflict of Interest Statement

The authors declare that the research was conducted in the absence of any commercial or financial relationships that could be construed as a potential conflict of interest.

## Supplementary Material

Supplementary Movie 1 Somite segmentation in Wilson explants was affected by CEP3-MO. Wilson explants receiving EGFP-CAAX on the left and EGFP-CAAX plus CEP3-MO on the right were imaged for 10 h at 10-min intervals. CEP3-MO affected cell shape changes and somite segmentation.
